# Containment and conversion: Urban livelihoods and the circulation of value amid South Africa's avian influenza outbreak

**DOI:** 10.1111/amet.13083

**Published:** 2022-06-24

**Authors:** THOMAS COUSINS, MICHELLE PENTECOST, LESLEY VAN HELDEN

**Affiliations:** ^1^ School of Anthropology and Museum Ethnography University of Oxford Department of Sociology and Social Anthropology Stellenbosch University; ^2^ Department of Global Health and Social Medicine King's College London/University of Cape Town; ^3^ Western Cape Veterinary Services Western Cape Department of Agriculture

## Abstract

In South Africa the racialized contours of economic life powerfully shape the distribution of who owns poultry enterprises, who is employed to labor in them, who consumes poultry products, and in which way. When, in late 2017, an outbreak of highly pathogenic avian influenza (H5N8) decimated the South African poultry sector, it revealed the ontological transformations of industrial egg‐laying poultry into “cull birds” and then into *imileqwa*, the quintessential rural chicken. It thus showed how distinct regimes of value “articulate,” blurring infectious and noninfectious concerns as new chains of conversion were inaugurated across domestic and global economies. Thanks to the mediations performed by the network of egg‐laying chickens, (White) farmers, (Black African) consumers, and state veterinarians, translations of value take place in which industrialized egg‐layer chickens turn into socially enlivened beings. Such beings sustain and nurture social reproduction in South Africa's postapartheid cities and beyond. [*zoonosis*, *value*, *human‐animal relations*, *global health*, *one health*, *race*, *urbanism*, *South Africa*]

“When the outbreak started in Zimbabwe, I thought, It's coming.” Dr. Erasmus's fatigue was palpable as he told the story.^1^ Our team of ethnographers sat with him in the Western Cape offices of the South African state veterinary services in September 2017. Earlier that year, the Western Cape Province had seen an outbreak of H5N8, a highly pathogenic avian influenza virus (HPAI). As a state veterinarian, Dr. Erasmus had tracked the first confirmed diagnosis when it appeared on a commercial farm in Zimbabwe's Mashonaland East Province, on May 17, 2017. By June the virus had spread to South Africa, appearing first on two commercial poultry farms in Mpumalanga Province, close to the northern border. Veterinarians and farmers in the Western Cape, on the southern tip of Africa, held a meeting within a week of the national alert; less than two months later the first positive cases of HPAI in the province announced themselves on ostrich, duck, and chicken farms. The farms were put under immediate quarantine. Within 24 hours of reporting the suspected outbreak, the first affected farm had recorded several thousand deaths. By the end of September, 13 commercial farms were affected, and 2 million layer hens had died.

Such a devastating loss of animal life and livelihoods reveals the delicate dependencies of South African postapartheid urban and rural economies on both local and intermediary forms of livestock production and consumption, as well as on globalized circuits of “lively” capital (Barua 2016). The arrival of H5N8 rippled through global and national commodity chains with very different effects for different actors. Picture what the sudden death of so many hens meant: as birds on White‐owned, egg‐laying farms died, farmers were panicked that their businesses would fail. The price of eggs skyrocketed. Economists worried that entire export sectors would be jeopardized. The sale of live chickens, for example, had halted abruptly. They normally flowed into secondary economies once their laying capacity was exhausted. As a result of their sudden scarcity, new supplies were desperately sought by the middlemen (they are almost all men) who buy exhausted layer hens to supply networks of roadside stalls. The roadside traders who sold these hens suddenly lost their livelihoods while families struggled to find affordable protein and did not have the live fowl crucial for rituals at key life events. By disrupting chains of production and consumption, the outbreak revealed forms of value beyond market assessments of the impact on egg sales. As this article demonstrates, these forms of value depend on the ontological transformations of live layer hens into *imileqwa*, popularly understood to be fowl raised in rural “African” homesteads. This value exchange brings together raced and classed categories of low‐income, Black African urban consumer; peri‐urban medium‐sized White farmer; and global circuits of lively capital. When the hens leave the farm, they are known as “cull birds”; when they are sold in township markets to Black African urban and rural consumers, they have been transformed into imileqwa, (even though it is difficult to stabilize definitions of “the rural” and “African,” and most imileqwa across the country are supplied by White‐owned industrial farms).^2^ By what means was this transformation effected, how was its importance revealed to state regulators, and what “values” emerged in the process?

Across South Africa, large supermarket chains dominate the foodscape, providing access to affordable food that is, however, low in nutritional value (Battersby 2017). In city “townships,”^3^food is also sold at roadside stalls, often adjacent to malls and other infrastructure (Tawodzera and Crush 2019). The roadside sale of food, including live fowl or imileqwa, forms an important livelihood strategy, often for women who migrate between rural areas and the cities, for example, between rural parts of the Eastern and Western Cape provinces and the city of Cape Town. The dearth of live layer hens, or “cull birds,” as they are referred to by farmers, was thus felt most acutely in township economies as a sudden loss of the availability of imileqwa.

*Imileqwa* is the term in isiXhosa (one of South Africa's 11 official languages) for fowl putatively raised in rural African homesteads, invested with the metonymic qualities of rural domesticity and personhood. Imileqwa resemble “chickens of Zuluness” (*izinkukhu zesiZulu*: free range domestic fowl), also known as “chickens of the people” (*izinkukhu zabantu*), as White (2011, 106) describes them in the context of KwaZulu‐Natal Province. These are not “chickens of Whiteness” (*izinkukhu zesiLungu*), the generic term for chickens produced through White‐owned industrial processes, which can be bought in supermarkets and which are also known as foolish, fake, or artificial fowl (*olamthuthu*) (White 2011). Discernibly different in size and color from commercially raised chickens, imileqwa are sold live, sometimes slaughtered at purchase for family meals or later for ceremonial purposes. Imileqwa are infused with the thick relations of domestic reproduction and serve as powerful agents of custom and ritual action in ceremonial and ordinary consumption. The H5N8 avian influenza virus thus ricocheted across finely calibrated value chains that turned on the specificity of imileqwa as a socioagricultural category and on its ontological transformations.

The drama of H5N8's arrival and the disappearance of imileqwa is driven home in the story of Xakathile, an energetic man in his mid‐40s who employed a team of 10 women to sell imileqwa in Khayelitsha, Cape Town's largest township, at four roadside stands. Amid the outbreak, Xakathile returned from a family funeral in Eastern Cape Province, a day's drive away, which had necessitated a substantial contribution of imileqwa for the ritual feasts that had accompanied the ceremony (Lee 2011). He usually sourced live chickens from egg farms on the edges of Cape Town. They were delivered by his supplier—known as a “cull buyer”—on Fridays, for sale during the weekend. In August 2017, with no live birds available, Xakathile could not continue to employ his usual helpers, and he sold out of the few imileqwa he could source by early Friday afternoon, leaving customers frustrated and angry ahead of weekend festivities.

As we tracked HPAI in the Western Cape in 2017, the outbreak made visible several distinct economies that came together around the availability of layer hens for the “cull market.” The argument developed here focuses on the conversion of “cull birds” into imileqwa, describing how the state, through its regulatory mechanisms, constructed the animal's ontology as it came into view and later fell apart. When distinct regimes of value converged, the imileqwa's importance emerged as an “ontologically multiple” point of conversion between biosecurity plans for containment, local economies of poultry consumption, and the history of racialized segregation of land, labor, and consumption.

At the heart of the state's efforts to contain the outbreak, there were two central concerns: First, the slippage in local understandings of *culling*, either as a state‐ordered public health measure to contain an outbreak, or as a strategy undertaken by farmers to economize on space, feed, and care (by, for example, selling “exhausted” egg layers). Second, the debate over the role of vaccination as a form of containment. Debates about “culling” and vaccination were shaped by the interests of different parties, including farmers, government officials, and live‐chicken traders. Should the state have rushed to vaccinate all poultry against H5N8 to prevent further spread, or was it too late? What might be the implications of vaccination compared to the mass extermination of birds? Should a vaccination campaign proceed in any case to prevent future avian influenza outbreaks? The outcomes of these debates had potentially far‐reaching implications for both local and global economies, given the fragility of food systems in South Africa and their thoroughly global interdependencies (Greenberg 2017). Moreover, these questions were closely tied to the maintenance, or closure, of the “cull bird” economy that delivered live layer hens to local traders—hens that were valuable both as imileqwa, with its ritual purposes, and as a protein source for poor South Africans.

In this article, we show how the arrival of the virus and the conundrum of how to contain it served as a revelatory vehicle for government officials and animal‐health professionals, one through which the transformation of birds was brought into focus, making legible race, class, and gender in the networks of production, consumption, labor, and value in postapartheid Cape Town. The question whether to vaccinate rested on a set of competing moral economies that brought together choreographies of biosecurity and crises of domestic reproduction in South Africa. The outbreak revealed the state's underpreparedness to deal with the complexity of avian influenza. It also showed how global arrangements of poultry, viruses, and trade policy are conditioned by local economies of consumption. In the South Africa context, the biopolitics of avian influenza was shown to be less a question of preparedness or surveillance than of confronting the persistence of the grammar of race, class, and the urban form that underpins poultry and other animal economies. The case we present contributes to a significant body of work on zoonotic outbreaks that has focused on preparedness, simulation, sentinels, and surveillance (Caduff 2015; Fearnley 2015; Keck and Lakoff 2013); here, we demonstrate the need to consider questions of regulation, trade routes, and the mundane afterlives of zoonotic outbreak.

## Tracking the outbreak

The outbreak of highly pathogenic H5N8 avian influenza (HPAI) during the Southern Hemisphere's late winter and early spring of 2017 ravaged the Western Cape's poultry industry with devastating economic and social effects for farms and urban livelihoods that were underappreciated in the outbreak's immediate aftermath (Khomenko et al. 2018). The virus had spread to sub‐Saharan Africa after an outbreak in the Northern Hemisphere's preceding autumn and winter, originating in Asia and spreading throughout western Eurasia and the Middle East, with sporadic outbreaks in India and Nepal (Sims et al. 2017). This was the most extensive HPAI epidemic ever documented; at its peak, H5N8 dispersed widely throughout the Afro‐Eurasian waterbird flyway system, and, as had been anticipated (Sims et al. 2016; Sims and Brown 2016), it reached Egypt and Tunisia in November 2016, followed in January 2017 by Nigeria, Niger, Cameroon, and Uganda. The virus most likely spread via the seasonal migration of wild aquatic birds, such as Palearctic dabbling ducks (Global Consortium 2016; Van den Brand et al. 2018). Wild birds migrating to Africa, however, sporadically introduced the virus, which became endemic in poultry and thus also spread across Africa's international borders through the poultry trade (Ekong, Fountain‐Jones, and Alkhamis 2018; Sims et al. 2016). In April–June 2017 the Democratic Republic of the Congo reported an accelerating H5N8 HPAI epidemic in poultry. In May, for the first time in the history of avian influenza observation, Zimbabwe reported having detected the virus. In June the virus was detected in South Africa, and by August 2017 it had arrived on farms in Cape Town. The 2017 epizootic outbreak was one of the worst of its kind since rinderpest ravaged cattle herds in South Africa in the late 1800s. State veterinarians estimated that 2.9 million chickens died or were culled in the Western Cape alone, including 2.8 million layer birds and 150 000 broiler breeders, constituting a sizable portion of the national losses of 5.4 million birds, including 4.7 million layers and 700,000 broilers (BFAP 2018). Production capacity decreased by 80 percent, and the price of eggs doubled. The total biological loss amounted to 317 million rand (US$17.5 million). Income lost from sales of eggs, pullets,^4^ chicks, and broiler meat was estimated at 1.5 billion rand, while the national losses for the poultry industry amounted to 1.87 billion rand (US$132 million) (BFAP 2018).

This article is based on fieldwork conducted from August to December 2017 by one veterinarian and two medical anthropologists (one of whom is also a medical doctor), each of whom has long‐term familiarity with human and animal health in South Africa and the Western Cape in particular. All three authors are, in South African terms, White and middle class, which surely structured our access and perspective in profound ways. We have sought to attend to the particular contours of race, class, and gender, our own and our interlocuters’. Although we undertook extensive ethnographic fieldwork in the locations we describe here, our situated perspectives necessarily obscure insight in some ways while assisting in others. Two of us are medically trained in South Africa, which helped us understand a range of health questions in social and administrative context. Two of us are women, which meant that we were particularly attentive to how gender shaped relationships between veterinarians and farmers, cull buyers and street traders, consumers and family ritual. We conducted participant observation in urban township economies with support from research assistants and conducted 20 interviews with state veterinarians, animal‐health technicians, government officials, imileqwa sellers, and consumers.

## Cull birds and the choreography of containment

In 2017 patterns of White farm ownership and Black labor continued to structure the poultry industry, reflecting a long history of racialized exploitation of cheap labor and inadequate occupational protections in South Africa's industrial and agricultural sectors (Packard 2016; Seekings and Nattrass 2005). Who in South Africa owns poultry enterprises, who is employed in the country's poultry industry, who consumes poultry products, and in which way—the distribution of these subject positions is powerfully shaped by the racialized contours of economic life established by a history of colonialism and then apartheid. Colonial governance before 1948, and then the system of laws and policies of apartheid, not only led to the collapse of an agrarian peasantry but also continue to shape urban space and consumption in postapartheid South Africa. The racialized geography of Cape Town remains profoundly shaped by the institutionalization in 1955 of the Coloured Labor Preference Area policy, which aimed to create a distinct “Coloured nation” in the Western Cape, according to Prime Minister Hendrik Verwoerd's secretary of native affairs, W. W. Eiselen (Goldin 1984). Black Africans were banned from living in Cape Town until 1983, when the policy was rescinded, but when the numbers of Black people settling in the Western Cape began to increase after 1983, apartheid spatial planning designated marginal land far from Cape Town for what the apartheid state designated “African” urban development (see endnote 2 for further explication of racial terms; Goldin 1984; Horner 1983). The policy of apartheid was designed to produce Whiteness as a legalized system of status, access to resources, and exclusionary civic rights for those classified as White. It also produced an immiserated Black proletariat that was forced to reside in “African homelands,” also known as Bantustans, which were designated for Black African people based on a putatively ethnic and spatial logic of difference (Sharp and Spiegel 1985). While displaced from White urban space, the apartheid system of migrant labor nevertheless forced Black Africans to find employment in cities, on mines, and on farms in White South Africa (Comaroff and Comaroff 1997; Murray 1987; Spiegel 1980).

Thus, the “choreography of containment” involves one key element from this history: namely, the racialized displacement of African populations and the policing of urban space. This partly explains contemporary patterns of poultry consumption in South Africa. Globally, the farming of broiler and layer hens are distinct industries. Broiler chickens are fowl bred solely to be sold in the meat industry. The layer industry produces eggs for commercial sale. It was this layer industry that was significantly affected by the 2017 H5N8 outbreak (BFAP 2018). On layer farms, hens produce eggs until the age of 65–75 weeks; then, when they have reached the end of their laying lives, they are “culled.” The *OED*’s general definition of the word *cull* is “to choose from a number or quantity; to select, pick.”^5^ But in the mid‐20th century a variation emerged from veterinary and wildlife practices in Australia, New Zealand, and the UK: “To select and kill (wild animals or birds), usually in order to improve the stock or reduce the population.” In the UK and US, culled layer hens are slaughtered en masse (Dixon 2002). In South Africa producers sell these layer hens to informal “cull buyers,” who distribute the live fowl to rural and urban consumers searching for putatively “rural” chicken—a wiry bird that satisfies the appetite for “real” chicken and that is also a requirement for many rituals. “They call them cull birds, but essentially what happens is that farmers sell those birds as live birds,” explained Dr. Ndlovu, a state veterinarian. “In the Western Cape, around half a million birds move off farms every month into the cull‐bird market. A large portion of them stay in the Western Cape and are eaten by people in the Western Cape. But a portion of them also get trucked to Gauteng and Kwazulu‐Natal, where demand is high.” The cull buyers, or middlemen, buy a few hundred or thousand birds at a time and transport them to a network of buyers across the country who sell live chickens from roadside stalls and backyard stores. The farmer recoups 25 rand per chicken (US$1.8 in September 2017), the cull buyer adds on his fee, and the consumer pays a premium for a live, authentic “rural” chicken, known in isiXhosa as *umleqwa* (pl.: *imileqwa*), perceived as tastier than commercially bought chicken and, within the symbolic economy of custom and tradition, infused with the qualities of rural domesticity. The dramaturgy of postrural livelihoods turns here on the crucial place of the homestead and its more‐than‐human members in postapartheid urban foodscapes.

Thus, in the context of the Western Cape's medium‐sized farms, to “cull” a bird can refer to *selling a live bird* to a cull buyer or to slaughtering a bird on the farm as a disease‐control measure. The state veterinarians explained that this slippage first became apparent during a *Salmonella gallinarum* outbreak in poultry in the Western Cape in 2016. As Dr. Erasmus recalled,

We had told farmers that their farms were under quarantine and that birds would have to be culled. But our understanding of “cull” and [farmers’] understanding of “cull” was very different: they wanted to *sell* the [live] birds. From their perspective, the longer they feed the birds, the less money [they make]. If they're feeding the bird and it's not producing an egg every day, then they're starting to lose money.

Not only does the sale of live birds recoup some costs, Dr. Erasmus explained, but it is also an essential part of the production chain. From the purchase of day‐old chicks that are raised on “rearing farms,” their move to laying farms at 18 weeks when they start to lay eggs, to their “cull” at 65–75 weeks, a tight choreography of production depends on this “cull” for financial viability:

You've got a house full of chickens and you've got chickens on order, and you only have a certain amount of time to get those birds out of there, clean the house, and get the new birds in, because the new birds are on the rearing farm until 18 weeks, and then they start laying eggs. You cannot make them hold off on laying eggs. Once they start laying, if they are on the rearing farm, it's a mess. It's literally a two‐year chain, a two‐year cycle. Those guys have ordered those birds a year and a half ago already.

For the farmers, the cull market thus has the dual function of helping recoup costs and, as part of the production chain, freeing up space for the next layer hens. For the cull buyers, the layer farms provide a stable supply of the *imileqwa* that their customers demand. When the outbreak began, these stakeholders’ conflicting needs were a key difficulty for state veterinarians, as was the mandate to contain the outbreak as a biosecurity priority. By August 2017, debates in the province about how to deal with an outbreak were in full swing, including the practical problem of how best to slaughter and dispose of large numbers of chickens, if necessary. In the immediate aftermath of the first positive HPAI cases, and a swift moratorium by the Department for Agriculture, Fisheries and Forestry on the selling of live layer hens from farms, the state veterinarians hosted a meeting attended by 80 cull buyers—a “flabbergasting” number for the state veterinarians, who had underestimated the size of this “local,” “informal” (and thus constitutively Black African) economy. While the existence of the cull‐buyer market had come to the attention of state veterinarians only in 2016, during the *Salmonella gallinarum* outbreak in poultry in the Western Cape, there had been no attempt to understand or regulate this crucially intermediating transaction. The veterinarian explained avian influenza, its potential impact on the industry, and the need for a moratorium on live‐bird sales. The audience was unsympathetic. “I need to feed my family!” one buyer shouted. “I need to buy birds tomorrow. When are they going to lift the ban on live cull birds?” Because imileqwa are a key feature of ritual events and feasts such as weddings, their sudden scarcity sent ripples through township economies (Petersen, Charman, and Kroll 2018). Suddenly, cull buyers had lost their sole product. With no live chickens for sale, consumers became angry. Dr. Lee, another state veterinarian, relayed the story of a cull buyer whose community threatened to burn her house down if he could not source imileqwa.

Divergent understandings of what it meant to “cull” were brought into focus by the difficulty of “choreographing” the containment of the virus. This concept of choreography draws on Thompson's (2005, 8) sense of the dynamic and uncertain coordination involved in the “deftly balanced coming together of things that are generally considered parts of different ontological orders (part of nature, part of the self, part of society).” Thus, for example, Law and Lien (2012) show how “farmed wild salmon” require a “careful choreography” that depends on precise arrangements of measurements, tools, and techniques. At stake in the avian influenza outbreak in Cape Town was the coordination of diverse elements “in highly staged ways so as to get on with the task at hand” (Thompson 2005, 8): namely, to contain the H5N8 virus. Farmers were instructed by state veterinary services to “cull” their birds: to slaughter and dispose of them en masse to limit further environmental contamination. Farmers now had to deal with the cost and effort required to slaughter birds according to regulations. They were instructed to inform state veterinary services how they were conducting the slaughter, to ensure that their methods were in line with the guidelines of the Society for the Prevention of Cruelty to Animals. Two methods of humane slaughter were acceptable: whole‐house gassing or manual cervical dislocation. Open‐air birdhouses are common in the Western Cape, which precluded whole‐house gassing. Manual cervical dislocation is time consuming and labor intensive, but it was the only option available to most farmers. Once the birds were slaughtered, they were to be disposed of by burial or composting. During the outbreak in Gauteng Province, farmers had tried burial, but it was space intensive, and veterinarians there had recommended composting to their colleagues in the Western Cape.

Composting is labor intensive, but it is an effective method of containing the virus, and it became the primary disposal method in the 2017 outbreak. While composting in the chicken house is preferable, for battery farms,^6^⁠ space was a limiting factor, so an area on the farm's periphery was instead designated for composting. The particular choreography of composting fowl was thus generally as follows: dead birds were loaded onto trucks parked as close to the poultry houses as possible to avoid contaminating the environment. The carcasses were distributed in two‐meter‐high heaps atop a base of straw, vine cuttings, or some other porous source of carbon. They were then covered by a thick layer of mature compost.^7^ As the composting took place, the temperature rose, which inactivated the virus and ensured that the compost could be safely used. State animal‐health technicians monitored the temperature of the mounds, which needed to stay at 55 degrees Celsius for three consecutive days. In sum, effective composting required the careful “choreography” of measurements, tools, and techniques for both pragmatic and communicative aspects of containment. Once the mounds were deemed safe, the compost could be sold to neighboring agricultural businesses, which helped farmers recoup some costs.

On paper, composting looks like an efficient and cost‐effective way to contain the outbreak. In reality, farmers were unfamiliar with the method and unhappy about the additional outlay required for composting materials and protective equipment. An operation that requires maximum efficiency and close monitoring was obfuscated by delayed visits from Environmental Affairs officials responsible for designating compost sites on each farm; by farms that failed to move dead birds fast enough; by farmers not purchasing the required materials; and by farm laborers’ unfamiliarity with the required techniques for culling, carrying, and composting birds. While animal‐health technicians were dispatched to farms to coordinate control efforts, they had to contend with wind or rain (which could disperse particles, lower the temperatures required to inactivate the virus, and interfere with the work itself); with the physical demands of monitoring heaps; and, in many cases, with farms that were inadequately equipped with basic biosecurity equipment, such as showers, gloves, and disinfectant (to protect against zoonosis and prevent humans from becoming vectors). Veterinarians feared the zoonotic infection of humans who either work in close contact with poultry or who slaughter their own poultry, through exposure to respiratory secretions and body fluids (OIE 2018). Animal‐health technicians had to train casual farm laborers in culling methods and how to use protective gear in the prescribed manner. The capacity for biosecurity was thus conditioned by the shortage of skilled labor, the lack of biohazard equipment, and changeable weather conditions.

Animal‐health technicians also became de facto counselors for desperate farm owners facing financial ruin. The economic implications of the cull were significant. For a family‐run medium‐scale farm (about 300,000 birds), a positive HPAI test was devastating. Efforts by farmers to slaughter infected birds may have been faster and more effective had a compensation plan been in place. While the law provides for farmers to be compensated, there were crucial delays in formulating a compensation plan, submitting it for approval, and implementing it. This caused much confusion and frustration among farmers and veterinarians. A key frustration for state veterinarians was the lack of clarity on whether farmers would be compensated for the birds they killed. “The whole crux of compensation is farmers are only paid for the birds that they kill. And they die fast, so farmers will start killing quickly, and then you get ahead of it,” Dr. Erasmus explained. The uncertainty about this meant that some farmers may have delayed destroying the birds in the hopes that they could sell live birds on the informal market and limit the cull's financial impact. In some cases, desperation led farmers to ignore mortalities in their birds, blaming the weather or a change in feed, and to alert the veterinarians only some days after the first deaths, thus delaying containment. As Dr. Snyckers, another veterinarian, explained, “You need to have a compensation plan before you have an outbreak. You cannot develop a compensation plan once you're in.”

Farmers were not always willing or able to hire additional labor to complete the slaughter quickly, which also prolonged the possibility of the virus spreading. Farmers also became suspicious, accusing each other of selling sick birds to cull buyers despite the moratorium on selling live birds since the outbreak had begun. “The system relied on everyone having integrity. So if nobody sells live birds, then you're fine. But if one person says, ‘Oh, here's a gap in the market,’ then everyone loses out,” Dr. Erasmus said. Farmers were also concerned that if they stopped selling to cull buyers, the buyers would simply go elsewhere, such as to Eastern Cape Province, where there was a perception that regulations were less stringently enforced.

Pressure from both farmers and cull buyers affected the state's institution of a permit system for the sale of live birds. While the moratorium was lifted, the supply of live chicken was severely curtailed once farmers acknowledged the severity of the outbreak and started slaughtering birds in the thousands in an effort to contain the spread. The outbreak revealed the extent and complexity of what had been an entirely unregulated market that was not only employing many people but also performing a social function and delivering cheap protein across the country. As one newspaper report put it, the outbreak meant that, on many counts, “more people will go to bed on empty stomachs” (Mehlwana 2017)—in particular, poor and working‐class Black African people.

The 2017 avian influenza outbreak had amplified and made apparent the difficulties of regulating the “cull market,” refracting the complex entanglements of race and class that shape the political economy of South African agriculture. In this context, the coded discourses of “commercial,” “emerging,” and “subsistence” farming index histories of race: “commercial” mostly denotes White‐owned, large‐scale farms; “emerging” points to Black farmers who receive state support for their commercial operations; and “subsistence” indicates farming for home‐use only. Since the 1990s, the postapartheid South African government's various agrarian and land reform policies have attempted to redress the racialized history of support for White farmers and the destruction of an African peasantry (Van Onselen 1996; Walker 2013). Terms such as *commercial* and *emerging* farms retain a perlocutionary force as codes for White‐ and Black‐owned farms, despite scholarly attempts to complicate such binaries (Cousins 2015). The choreography of the cull took place against this charged backdrop of raced and classed labor relations on commercial farms. These relations complicated efforts to contain the virus, to assist farmers, and to mitigate the outbreak's larger economic impacts. At the center of these intersecting concerns about biosecurity and food security lay an important question about the place of vaccination in halting and preventing avian influenza.

## Vaccination and value

Farmers and veterinarians alike expressed helplessness as they watched the outbreak ravage the Western Cape poultry industry. Farmers insisted that the government “should do something.” But what could be done about an airborne virus that mutates frequently and is probably transmitted via migratory wild birds or waterfowl? (Fearnley 2018; Scoizec et al. 2018). With pressure mounting on officials to offer *something* to the affected farmers, discussions began about vaccination. For farmers, vaccination seemed like a good option: vaccinate future stock to prevent another outbreak, rather than destroy all birds and lose income. In a meeting with stakeholders, farmers asked the provincial minister to discuss with the national government.

State veterinarians were less enthusiastic about how effective vaccination might be and were concerned about its implications. “I understand the decisions [that politicians] make—to save themselves—but vaccination is not a silver bullet,” Dr. Erasmus said. “You have to be prepared for the level of biosecurity associated with [vaccination], and government needs the capacity to manage vaccination, and quite frankly, we don't have that capacity now.” A colleague explained the complexity that vaccination introduced: “The national department will consider allowing it if you're prepared to have your layer hens slaughtered at the abattoir [for safe disposal, at farmers’ expense] at the end of their production. You will not be allowed to sell live birds into the cull market.” According to international biosecurity standards that have developed since the 1960s, but in particular since the 1997 outbreak of avian influenza in Hong Kong, any vaccinated bird must be slaughtered in a registered abattoir (OIE 2018). It would no longer be possible to sell birds live in any market, because a vaccinated bird might carry HPAI but not manifest clinical symptoms while possibly shedding virus. Farmers and cull buyers were unwilling to accept those conditions. Farmers were quick to compare the situation to the 1970s outbreaks of virulent Newcastle disease, which is caused by another avian virus transmissible to humans, and which was effectively eliminated through vaccination. The veterinarians stressed that, unlike Newcastle, the influenza virus mutates quickly, and it is impossible to vaccinate against all strains. In addition, they stressed the impact of vaccination on the country's export status. Resorting to vaccination would imply that avian influenza was becoming endemic, threatening food and animal safety standards required for export. In the 1970s, South Africa was excluded from global markets by international sanctions against apartheid, so vaccination was not a concern for the poultry sector. To vaccinate in 2017 would threaten the country's export status, with significant economic ramifications, including vulnerability to cheap imports from China, the United States, and Brazil.

The poultry industry in South Africa is the largest segment of the agricultural sector, contributing more than 20 percent of its share of GDP and 43 percent of animal product GDP (SAPA 2018). Thus, secure access to global markets was crucial to the industry's viability and to South Africa's agricultural export economy more broadly. Vaccination would switch the country's disease status to “endemic,” with consequences for surveillance and export certification. Unregulated vaccination would in effect be seen as a proxy for the country's failure to properly control the virus. If the virus was understood as “endemic,” that is, as consistently present in a given population or area, and not a visitation from without, poultry and related products could not be exported because they posed a danger to other regions. Clearly, what is “endemic” is less a straightforward epidemiological fact than a biopolitical relation established through a country or territory's measures against, and capacity to control, disease and death (Antia and Halloran 2021; Servitje and Nixon 2016). While a range of national responses to avian influenza have been demonstrated, such as in China, Hong Kong, Vietnam, and the UK, the tension between vaccination and culling was perceived as the only available choice in South Africa.

Farmers and veterinarians were thus at an impasse: a vaccination program might prevent further outbreaks, but this could have deleterious effects on the cull market and the country's export prospects. While farmers thought vaccinating would allow them to continue selling live birds, veterinarians worried that a regulatory threshold would be crossed, namely that the virus would be perceived as uncontained, and thus on its way to “endemic” status, with far‐reaching effects beyond the domestic economy. As one veterinarian put it, “On vaccination, we're at checkmate.”

According to Dr. Erasmus, farmers resisted advice from veterinary services largely because they lacked knowledge about the disease, biosecurity methods, and regulatory standards. A more complex picture, however, is suggested by the long history of tense relations between state veterinarians and farmers in South Africa (cf. Beinart and Brown 2013; Mather and Marshall 2011; van Helden et al. 2016). As Dr. Erasmus put it,

They don't understand how diseases work, and that's an indictment on vets. I don't think we have done enough to educate farmers about diseases, but you also get these difficult farmers. So when the government says that in order to vaccinate birds, they will have to be slaughtered [i.e., at a certified abattoir, under strict biosecurity controls], then they bring their cull buyers and say that won't suit them. At the end of the day they're saying that they don't want to change a single thing about the way they farm. They don't want to improve biosecurity. They don't want to slaughter their birds. But they want government to allow vaccination because they are not going to place birds on the farm again unless they can vaccinate them because the risk is too high.

Farmers wanted the insurance of vaccination without taking up the additional biosecurity measures (such as slaughter and composting under controlled conditions) and without the loss of cull‐bird sales that this would entail. Vaccination appealed to farmers and government officials who wanted action. Dr. Erasmus empathized with the farmers’ situation. “There is this recognition that farmers take on a certain amount of risk when they farm,” he said, “but if that risk becomes too high, then nobody's going to farm, and then who is going to feed our nation?”

Yet vaccination would have far‐reaching implications for both local and global poultry economies. Vaccination would mitigate farmers’ short‐term risk of losing further stock but keep them from recouping their costs, because they could no longer sell live birds to cull buyers.

Those in search of cheap protein would also face the nutritional consequences of the poultry's unavailability—in 2017, imileqwa cost 25 rand a bird (US$2.50) at more accessible roadside stalls, while supermarket broilers cost 45 rand (US$4). For those hoping to shore up the relational values of such lively commodity forms, other candidates for imileqwa would have to be found. At a global level, vaccination would condition certain spheres of exchange by switching South Africa's disease status to endemic and thereby threatening export status, with macroeconomic effects on food prices and food security. Conversely, vaccination would enable other spheres of exchange: cheap imports of (processed) chicken would flow more readily into the South African market (e.g., Booysen 2019), making for cheaper fast food but undermining the domestic (i.e., national) poultry sector, already embroiled in a trade war with the US (Speckman 2018). On the contrary, opting not to vaccinate would preserve the country's export status and the cull‐bird economy. In sum, the debate over vaccination brings into focus two points of “conversion” of value (Guyer 2004) that make visible different ontological practices and moral economies in the struggle to contain avian influenza in South Africa. To trace these conversions, one needs to consider a set of ontological questions about how birds are constituted, as well as a set of political questions about the production of social subjects in historical‐materialist terms.

## Conversions of value

Guyer (2004, 30) defines a conversion as what “adds, subtracts, or otherwise transforms the attributes of exchange goods in ways that define the social direction of future transactional possibilities.” Building on Bohannan's (1955) notion of Tiv “spheres of exchange,” Guyer expands Bohannan's original three spheres of exchange into an open‐ended chain of historically constituted transactions that, in the words of Bourdieu (1977, 195), afford the “endless reconversion of economic capital into social capital.” An important conversion is at work in the transformation of exhausted egg‐laying hens into imileqwa, the quintessentially rural “African” chicken that animates township food and ceremonial economies. The ontological mutability of “cull birds” that existed before the outbreak thus articulated several different regimes of value that, in the context of the 2017 outbreak, began to blur infectious and noninfectious concerns as new chains of conversion were inaugurated across domestic and global economies. These racialized conversions of animal kinds created an impasse between opting for mass vaccination and declining to carry it out. Opting for vaccination (at the state's expense), together with the regulated slaughter of poultry (at the farmers’ expense, with the subsequent loss of income from cull sales), might prevent future avian influenza outbreaks, but it would threaten local livelihoods and the country's export markets. Opting not to vaccinate, on the other hand, would preserve local economies and global export status, but would leave the industry vulnerable to another devastating outbreak in the future. The decision to vaccinate would have decimated the cull market and provoked a profound shift in export and domestic food economies, with long‐term implications for ceremonial and secular domestic consumption, and the availability of cheap protein for impoverished communities. As it turned out, chickens died faster than they could be slaughtered or vaccinated, and farmers had to compost anyway, with dire consequences for business viability.

For Guyer, conversions involve the directional transactions that constitute stores of value (see also Comaroff and Comaroff 1990). The South African outbreak of HPAI brought together several forms of value, alongside the thickening of social relations around the consumption of the desirable, ritually efficacious, rural chicken; the provision of cheap protein to a food insecure population; and the country's retention of its export status for poultry products, thereby preventing an influx of cheap imports and protecting local economic activity. Thus, one might say that the state's attempt to contain the outbreak conjoins the body of the chicken and the body of the consumer through conversions of value, with material, semiotic, and metabolic effects for both human and fowl. In this case the H5N8 virus (or viruses) articulates global geopolitics and local economies and reveals the nutritional and relational stakes at the heart of these networks. By shifting away from seeing chickens as naturalized objects toward a more relational and performative construction, one can better apprehend the practices and capacities that must be choreographed to produce particular effects (such as value, labor, eggs, or “rural” chicken). Imileqwa operate as a sign between several systems of meaning, exceeding the “bounded and localized system of meanings” (Appadurai 1986, 15) that commodity exchange traverses. Regulated poultry markets are interdependent with racialized, apparently unregulated, so‐called informal markets for live birds in Cape Town, but before the H5N8 outbreak this conversion of value between articulated economies of production and consumption had been invisible to state regulators and global health and trade regimes. The mediations performed by the network of egg‐laying chickens, (White) farmers, (Black African) consumers, and state veterinarians, effect translations of value that turn industrialized egg‐layer chickens into socially enlivened beings that sustain and nurture social reproduction in postapartheid South African cities (and beyond).

Since the democratic transition of 1994 and South Africa's reintegration into the global economy, the circulations of commodities, plants, animals, and viruses has pulled into focus the intimate entanglements between capital, human and nonhuman forms of life, and ideas about race and ethnicity (Comaroff and Comaroff 2009; Foster 2017). Along with growing anxieties about alien species and zoonotic pandemics, new global regulatory efforts have sought to keep humans and nonhumans in their proper places (Helmreich 2009). Thus, as impure actors embodying postapartheid and globalized forms of value, imileqwa are constitutively tied to global circulations of avian influenza through a series of conversions in which sentinels and surveillance are brought together with thick ethical concerns with compensation and food security across national, regional, and global networks. Imileqwa are forms of life through which social ties and enduring bodies might be nourished. Ontologically, the conversion of layer hens into cull birds into imileqwa occurs via a series of relational performances informed by racialized histories of farm labor, the spatial segregation of South African cities, the articulation of economies, and the reconstitution of the rural in urban life.

Such relational performances are central to grounded responses to a crisis of personhood in South Africa, in which older forms of becoming full persons through gendered migration have become impossible, and inventive responses have been required (Ferguson 2013; White 2012). White (2011) skillfully shows how local moral economies of animal kinds engage that crisis of personhood; similarly, Porter's (2013, 132) work in the Vietnamese context shows how concerns about biosecurity are subject to “heterogeneous moral codes surrounding animals’ role in knowledge hierarchies, village economies, and notions of individual worth,” human and nonhuman. White (2011, 105) argues that “relations with animals function not as indices of given cultural differences, but rather as social practices aspiring to create particular forms of differentiation.” By taking animals’ entanglements in human affairs as vectors for the constitution of different social ties, White seeks to understand what the ascription of racial or ethnic labels to animals, for example, of cattle and chickens as beasts of “Zuluness” or “Whiteness,” reveals in contemporary KwaZulu‐Natal about the management of social difference. Eschewing symbolic/culturalist reductions of animals as merely good to think with, and reaching for a stronger materialist reading of animals as media for the pragmatic constitution and mediation of social relationships, White asks how animals enter into the making and marking of differences in human affairs. White's ethnography lays out the categories that we found to be alive to cull buyers and consumers in Cape Town's poultry economies: imileqwa are precisely this ideal type, namely the homestead chicken that is tasty, healthy, and thickly entailed in social relations. The broiler industry does not produce “waste” that can be recirculated in new forms of value, while spent layer hens are available for transformation via resale in unregulated markets. Here the racialized history of labor migration, in which so many Black Africans were forced to move between so‐called homelands to urban centers of work, comes to matter in new ways, as a different set of (nonhuman) bodies are set into motion, along new metabolic pathways (Murray 1987; Newell and Cousins 2014; Porter 2019; Watts and Goodman 1997). Why it is that “cull birds” should be the prime actor eligible for transformation into imileqwa speaks as much to the collapse of postapartheid “peasant” agrarian economies as it does to the dynamics of urban informal economies and, of course, their close interrelationship (Du Toit 2017; Hornby et al. 2017).

To understand the mundane aftereffects of the zoonotic epidemic in this case, it is necessary to revisit the role of animal kinds in the making of persons in postapartheid South Africa. Spent layer hens, sold alive to be sold again as imileqwa, occupy an intermediary space between industrial and rural chicken, akin to the indeterminacy of the categories of wild and domestic observed by Fearnley (2018), but they also effect an “investment” of the kind Guyer (2004) discusses in Bohannon's work on Tiv spheres of exchange. The HPAI outbreak showed how township and rural domestic and symbolic economies are profoundly articulated with national poultry and egg production systems, which are in turn shaped by geopolitical battles for trade access and global health concerns with zoonosis (Breitenbach 2019). In White's (2011, 107) terms, imileqwa and industrial broilers have “different kinds of flesh, and these produce dissimilar effects in the lives of humans who eat them.” In the context of avian influenza in Cape Town, these processes, relationships, and connections are powerfully shaped by the historical legacies of race‐based social engineering, the vicissitudes of global economies and global health logics of preparedness, and imaginative efforts to constitute persons.

## Conclusion: The ordinary afterlives of a zoonotic outbreak

The significance of the H5N8 outbreak was not only that it provoked a tension between biosecurity on the one hand and urban livelihoods on the other; more importantly, it made legible to the state the articulation of distinctly racialized regimes of value and consumption that draw on enduring patterns of racialized difference (Gillespie 2014) and the persistent relationship of capital to race in postapartheid South Africa (Dubow 2017; Wolpe 1972; Wolpe 1988). The crisis of the 2017 H5N8 outbreak and the uncertainties over culling and vaccinating revealed existing chains of conversion that link personhood, domestic reproduction, animal life, nutrition, and global trade in concatenating relations. Tracing conversions of value revealed how imileqwa, as ontologically unstable and multiple, disturbed the choreography of biosecurity and the regulatory logic of global health.

If, as Keck (2018b) suggests, anticipatory biosecurity reveals the instabilities of zoonotic pathogens and their effects on human sociality, then avian influenza in South Africa requires a different perspective on the social. It is in charting the mundane afterlives of an outbreak that translations of value between and across forms of life and their commodity forms become visible. In following the “ordinary” translations of value entailed by HPAI (Keck, Kelly, and Lynteris 2019), we can ask how “living with biosecurity” might engender new ways of learning to recognize and respond to emerging epizootics. Since the 1990s, biosecurity has become a normalized public concern and modality of global health (as a “human” concern; Packard 2016) and the One Health initiative (which attempts to integrate human, animal, and environmental health matters; Cassidy 2018). Biosecurity is now a potent force in contemporary geopolitics (Keck and Lynteris 2018; Lakoff 2017; Mather and Marshal 2011). Thus, in the context of enduring arrangements of race and more‐than‐human forms of life in postapartheid South Africa, the H5N8 outbreak has forced a reckoning with pandemic preparedness and the everyday life of political and moral economies of consumption (Adebanwi 2017).

Influenza outbreaks become a lens for understanding how ideas about viruses and race interact. HIV in South Africa has a distinctly racialized distribution that informs popular ideas about racial difference (Mabaso et al. 2019; Nattrass 2013; Packard and Epstein 1991), and Euro‐American anxieties about Ebola provoked powerfully racialized responses to the outbreak, as Monson (2017) shows. Where H1N1 influenza in China was understood to be shaped by racial differences between Mexicans and Americans, in the first instance, and then ethnonationalist distinctions between Han and other Chinese groups (Mason 2015), H5N8 in South Africa did not disturb existing racialized economies, nor was the virus itself understood to affect racialized bodies differently. Rather, the virus made legible enduring economies of production and consumption that remain profoundly connected through the racialized differences between who owns chicken farms and who purchases live layer hens, and why. The lively intervention of the avian influenza virus both reveals and transforms these articulated networks by putting into question the regulations, habits, rituals, and norms of global chick suppliers, local egg producers, veterinary officials, and unregulated local poultry economies. Thus, the question whether to vaccinate illuminates points of conversion of value between distinct moral economies that include global biosecurity, national export status, and domestic reproduction. In this case there were two opposing opportunities for conversion: to vaccinate or to preserve a cull‐buyer market. Either would enact a different moral economy and constrain or enable divergent chains of conversion.

In tracing how culling, vaccinating, and consuming came into view through the outbreak, the instability of chickens also reveals the instability of infection as a category of analysis. The extensive anthropologies of HIV, Ebola, and malaria, among other infectious diseases, suggests that instead of assuming the universal meaning of infectiousness, we should attend to how particular viral agents enter into human affairs, shaped by ideas about matter‐out‐place (Douglas 1966), cultural sensibilities, and moral responsibility (Lakoff, Collier, and Kelty 2015). The story of the struggle to secure access to antiretroviral treatment for HIV in South Africa is one such example (Cousins 2015; Geffen 2010). Such instabilities of infection are particularly evident in avian influenza (Fearnley 2015; Keck 2020; Porter 2019). It is no longer the virus itself that is in question (and studied under microscopes), but rather influenza's milieu that is critically in question, thus locating pathology in ecological and multispecies arrangements; this is what anthropologists have called a “biology of context” (Caduff 2012, 344), at the “frontiers between species” (Keck 2014, 59), or amid a “multispecies cloud” (Lowe 2010). Indeed, much of this literature takes inspiration from Latour's (1988) analysis of pasteurization, in which diverse actants are brought together across scales of space and time, mediated by technologies and techniques, and situated within broader social and political currents. Keck's (2018a) historiography of animal diseases and social anthropology takes this point a step further, suggesting a structural relationship between the social demands of zoonotic outbreaks and the kinds of concepts and analyses developed in the science of the social to respond to and contain them.

In the case of Cape Town's 2017 avian influenza outbreak, the (dis‐)articulations of infection, vaccination, and culling point to the instability and mobility of the meanings of infection across registers of value and forms of life (cf. Mavhunga 2011). Not only that, but vaccination paradoxically brings into articulation infectious and noninfectious disease models in a time of zoonotic outbreak and nutritionally related chronic illness. The conversions of value at stake in these encounters move across categories of communicable and noncommunicable disease, stitching together viral logics of preparedness and containment with more mundane crises of everyday sustenance. As Seeberg and Meinert (2015) have argued, the medical distinction between infectious and noninfectious diseases does not align with public health's distinction between “communicable” and “noncommunicable,” and thus confuses debates over the definition of what constitutes an epidemic. Their concept of an “analytics of biosocial epidemics” has purchase here, where the concept of contagion shifts from one that is focused on essence and substance to a broader notion that includes processes, relationships, and connections.

Scholars should pay attention to ordinary sensibilities of taste, the play of animal kinds, and situated struggles to secure nutrition and well‐being. Doing so is vital to apprehending the ontological transformations entailed in ordinary, multispecies economies. As the 2019 SARS‐COV2 outbreak has again demonstrated, Euro‐American sensibilities regarding which animals should or should not be sold or eaten, in which contexts, and by whom, draw on deep reservoirs of cultural investment in categories of purity and danger (Douglas 1966), geopolitical competition (Friedman 2020), and global health capacity (Hui et al. 2020). As Mather and Marshall (2011) suggest, we need alternative approaches to analyzing biosecurity that avoid the flattening effects of global biosecurity on local knowledge. We have sought here to open up the importance of ontologically multiple and mutable forms of life and their articulations of value across distinct points of conversion. We have done so to show how global biosecurity operates as one concern among others in the management of zoonotic outbreak and its ordinary afterlives.

## Notes

1 All interlocutors’ names are pseudonyms.

2 In this article, we use the capitalized terms *White* and *Black* to indicate the constructed nature of these racial categories (Goldberg 2009; Mbembe 2017). The postapartheid South African national census uses the categories “black African,” “white,” “coloured,” and “Indian/Asian,” which also appear in this article (SSA 2019). At different points during the 20th century, the state used different classificatory terms for racial categories. One of the more infamous pieces of apartheid legislation, the Population Registration Act of 1950, decreed that each citizen should carry an identity document that recorded their race as either “white,” “coloured,” or “native”; this was later modified to divide the population into four groups: “African,” “Indian” “Coloured,” and “White” (Posel 2001). The anti‐apartheid liberation movements used a more expansive conception of “Blackness” to build solidarity among all those discriminated against by the apartheid system and thus included “coloured,” “Indian,” and “Asian” people in this political category. In this article, the term *coloured* refers to the South African census category for people of mixed‐race descent, and we retain this spelling to mark the specificity of term.

3 “Township” refers to an urban area previously designated for Black African residents during apartheid. In postapartheid Cape Town urban space and practice cannot be separated from the long history of racial segregation, and township residents are still predominantly Black African (Gillespie 2014).

4 A pullet is a young hen under one year of age that has not started laying eggs yet.

5
*Oxford English Dictionary*, s.v. “cull, n.3,” accessed April 24, 2022, https://www.oed.com/view/Entry/45649.

6 Battery cages are a housing system used for various animal production methods, but primarily for, egg‐laying hens. The name arises from the arrangement of rows and columns of identical cages connected together, in a unit, as in an artillery battery. Although the term is usually applied to poultry farming, similar cage systems are used for other animals. Battery cages have generated controversy between advocates for animal rights and industrial producers.

7 Mounds were covered with plastic in some cases to prevent wild birds from scavenging the dead chickens, but leaving the mounds uncovered is apparently better for the composting process, which requires air flow for a supply of oxygen through the heap.
